# Mean-Square Radius of Gyration and Scattering Function of Semiflexible Ring Polymers of the Trefoil Knot

**DOI:** 10.3390/polym8080271

**Published:** 2016-07-27

**Authors:** Hiroki Abe, Daichi Ida

**Affiliations:** Department of Polymer Chemistry, Kyoto University, Katsura, Kyoto 615-8510, Japan; abe@molsci.polym.kyoto-u.ac.jp

**Keywords:** semiflexible polymer, ring polymers, trefoil knot, mean-square radius of gyration, scattering function, Monte Carlo simulation

## Abstract

A Monte Carlo study of the mean-square radius of gyration $R_{g}^{\;2}$ and scattering function P(k) with *k* the magnitude of the scattering vector for semiflexible ring polymers of the trefoil knot was conducted by the use of the discrete version of the Kratky–Porod (KP) wormlike ring model. The behavior of $R_{g}^{\;2}$ and P(k) as functions of the reduced contour length λL, defined as the total contour length *L* divided by the stiffness parameter $\lambda^{-1}$, is clarified. A comparison is made of the results for the KP ring of the trefoil knot with those for the KP ring of the trivial knot and for the phantom KP ring without the topological constraints.

## 1. Introduction

A vast amount of experimental, theoretical, and computational results have been reported for the dilute solution properties of *flexible* ring polymers, where a comparison was made of experimental and/or computational results with conventional Gaussian chain theories [1,2,3]. On the other hand, only a few studies have been made for *semiflexible* rings [4,5], where experimental data obtained for circular DNA in a limited range of molecular weight *M* were analyzed by the theory on the basis of the Kratky–Porod (KP) wormlike [5,6] ring model.

In order to obtain a deeper understanding of the effects of chain stiffness on the dilute solution behavior of ring polymers, we conducted Monte Carlo (MC) studies of the dilute solution properties, such as the second virial coefficient $A_{2}$ at the Θ state [7], scattering function P(k) with *k* the magnitude of the scattering vector [8], and intrinsic viscosity [η] [9], of semiflexible ring polymers by the use of a discrete version [5,10,11] of the KP ring. There, the behavior of these quantities as functions of the reduced contour length λL [5] has been clarified, λL being proportional to *M* and defined as the total contour length *L* measured in units of the stiffness parameter $\lambda^{-1}$ [5], in the range of the crossover from the rigid-ring limit λL→0 to the random-coil one λL→∞. We note that $\lambda^{-1}$ is equal to twice the persistence length (or to the Kuhn statistical segment length) as far as the (continuous) KP model is concerned [5]. It has been pointed out that even for ring atactic polystyrene (a-PS), a typical flexible ring with large *M* ($∼10^{5}$) or λL ($∼10^{3}$), the effects of chain stiffness are still remarkable. Further, Terao et al. [12] has recently shown that the experimental data of $A_{2}$ at Θ for cyclic tris(*n*-butylcarbamate)—which is a novel and typical example of semiflexible ring polymers—agree fairly with our MC results.

Equilibrium conformational properties of ring polymers may be affected not only by the effects of chain stiffness but also by those of the intramolecular topological constraints, which work to preserve the type of knot for a given ring polymer. We have also examined the effects of constraints on $A_{2}$ [7], P(k) [8], and [η] [9], along with the mean-square radius of gyration $R_{g}^{\;2}$ [7]. Specifically, a comparison has been made of these quantities evaluated for the KP ring of the trivial knot (unknotted KP ring) with those evaluated for the KP ring without the topological constraints (phantom KP ring). It has been shown that the difference in the quantities between the two kinds of KP ring becomes more and more remarkable with increasing λL in the range of λL≳10, while for λL≲10 the difference is negligibly small.

Considering the fact that for (semiflexible) circular DNA, the configurations not only of the trivial knot but also of non-trivial knots are visualized by electron microscopy [4,13], and that liquid chromatography at critical condition makes it possible to separate the (flexible) ring a-PS of non-trivial knots from those of the trivial knot [14], it is necessary to investigate the dilute solution behavior of both semiflexible and flexible ring polymers of non-trivial knots for the fine characterization of ring polymers. Such examination has been made theoretically and/or computationally only for flexible rings by the use of the Gaussian ring model or the corresponding models [15,16,17,18,19,20,21], as mentioned generally above. In this paper, we examine the effects of chain stiffness on the dilute solution behavior of ring polymers of non-trivial knots. For simplicity, we focus on the unperturbed rings (without excluded volume) of the trefoil knot (or 3${}_{1}$ knot in the Alexander and Briggs notation [22,23]), which is the simplest non-trivial knot and may be considered as the majority in the above-mentioned ring a-PS samples of non-trivial knots, because their *M* is not very large [$O(10^{5})$]. The most fundamental quantities $R_{g}^{\;2}$ and P(k)—reflecting the repeat-unit distribution around the center of mass of a single polymer in solution, determined by light scattering or small-angle X-ray or neutron scattering measurements—in the field of polymer solution science are evaluated by the MC method using the ideal discrete KP ring of the trefoil knot, and their behavior is examined as functions of λL with comparison between the present results and the previous ones for the KP ring of the trivial knot and for the phantom KP ring.

## 2. Model and Method

The present MC model and method are the same as those in the previous studies [7,8,9]—i.e., a discrete version of the KP ring proposed by Frank-Kamenetskii et al. [5,10,11], except for the construction of the statistical ensemble of configurations of the trefoil knot. The discrete KP ring is composed of *N* junction points connected by *N* bonds of length *b*. Let $b_{i}$ (i=1, 2, ⋯, N−1) be the *i*th bond vector from the *i*th point to the (i+1)th. The *N*th bond vector $b_{N}$ completes the ring; that is, $\sum_{i=1}^{N}b_{i}=0$. The configuration of the ring may then be specified by the set $\left\{b_{N}\right\}=\left[b_{1},b_{2},\cdots ,b_{N-1}\left(,b_{N}\right)\right]$ apart from its position and orientation in an external Cartesian coordinate system. Note that $b_{N}$ is a dependent variable for the ring. The configurational energy *U* of the ring has been given in terms of the angle $\theta_{i}$ (i=2, 3, ⋯, *N*) between $b_{i-1}$ and $b_{i}$, and $\theta_{1}$ between $b_{N}$ and $b_{1}$, as follows [5,10,11],

(1) $$U\left(\left\{b_{N}\right\}\right)=\frac{\alpha }{2}\sum_{i=1}^{N}\theta_{i}^{\;2}\;$$

where βα is the bending force constant with *β* the reciprocal of the product of the Boltzmann constant $k_{B}$ and the absolute temperature *T* [24]. This model may be regarded as the freely rotating ring with bond angle supplement $\hat{\theta }=\arccos\langle \cos\theta \rangle$, where 〈cosθ〉 is defined by

(2) $$\langle \cos\theta \rangle =\int_{0}^{\pi }e^{-\beta \alpha \theta^{2}/2}\cos\theta \sin\theta \;d\theta /\int_{0}^{\pi }e^{-\beta \alpha \theta^{2}/2}\sin\theta \;d\theta \;$$

We note that the MC model reduces to the freely jointed ring in the limit of α→0.

Although the pesistence length $L_{p}$ *as a discrete model* for this ring may be given by $L_{p}=b/\left(1-\langle \cos\theta \rangle \right)$, we introduce another measure of chain stiffness in order to maintain consistency between the MC data analysis in this study and the analyses of experimental and/or computational data on the basis of the contiunuous polymer chain model, as done in the field of polymer solution science [5]. We consider the (continuous) linear KP chain of contour length *L* and of persistence length *q* *as a continuous model* or stiffness parameter $\lambda^{-1}=2q$ [5], which reproduces the behavior of the mean-square end-to-end distance $R_{\mathrm{ee}}^{\;2}$ (or $R_{g}^{\;2}$) of the linear freely rotating chain of number of bonds *N*, bond length *b* (total backbone length Nb), and bond angle supplement $\hat{\theta }$ (=arccos〈cosθ〉) under the restricition L=Nb. Considering the fact that $\lim_{L\to \infty }R_{\mathrm{ee}}^{\;2}/L=2q=\lambda^{-1}$ for the former chain, and $\lim_{L\to \infty }R_{\mathrm{ee}}^{\;2}/L=b\left(1+\langle \cos\theta \rangle \right)/\left(1-\langle \cos\theta \rangle \right)$ for the latter, it is then necessary to equalize $\lambda^{-1}$ with b(1+〈cosθ〉)/(1−〈cosθ〉),

(3) $$\lambda^{-1}=2q=b\frac{1+\langle \cos\theta \rangle }{1-\langle \cos\theta \rangle }\;$$

It is seen from Equation (3) that $\lambda^{-1}$ as a continuous model so defined is equal to the product of *b* and the characteristic ratio $C_{\infty }=\left(1+\langle \cos\theta \rangle \right)/\left(1-\langle \cos\theta \rangle \right)$ as a discrete model, and becomes identical with $2L_{p}$ in the limit of 〈cosθ〉→1 (α→∞). In this study, with regard to the discrete KP ring (the freely rotating ring of bond angle supplement $\hat{\theta }$) as the continuous KP ring of stiffness parameter $\lambda^{-1}$ calculated from Equation (3), we make an analysis of the MC data on the basis of $\lambda^{-1}$ (or *q*) instead of $L_{p}$.

For the initial configuration $\left\{b_{N}\right\}$, we adopt an *N*-sided regular polygon of side length *b*—which is the most stable configuration—and sequentially deform it by the virtual motion introduced by Deutsch [25]. Let **v** be the unit vector along the vector distance between a pair of joints randomly chosen under the condition that they not be next to each other. If the *i*th and *j*th joints (i<j) are chosen, **v** is along the vector sum $\sum_{k=i}^{j-1}b_{k}$. A trial configuration $\left\{b_{N}^{\prime }\right\}$ is generated by rotating the shorter part of the ring around **v** by an angle *ϕ* randomly chosen in the range of [−π,π). The bond vectors $b_{i}$, $b_{i+1}$, ⋯, $b_{j-1}$ are rotated if j−i≤N/2, and the rest otherwise. If the bond vector $b_{k}$ undergoes the rotation, $b_{k}^{\prime }$ may be given by

(4) $$b_{k}^{\prime }=vv\cdot b_{k}+\left(\cos\phi \right)\left(I-vv\right)\cdot b_{k}+\left(\sin\phi \right)v\times b_{k}\equiv R\left(v;\phi \right)\cdot b_{k}\;$$

where **I** is the unit matrix and the rotation matrix R(v;ϕ) is given by

(5) $$R\left(v;\phi \right)=\left(\cos\phi \right)I+\left(1-\cos\phi \right)\left(\begin{array}{ccc} v_{x}^{\;2} & v_{x}v_{y} & v_{x}v_{z}\\ v_{y}v_{x} & v_{y}^{\;2} & v_{y}v_{z}\\ v_{z}v_{x} & v_{z}v_{y} & v_{z}^{\;2} \end{array}\right)+\sin\phi \left(\begin{array}{ccc} 0 & -v_{z} & v_{y}\\ v_{z} & 0 & -v_{x}\\ -v_{y} & v_{x} & 0 \end{array}\right)$$

with $v_{x}$, $v_{y}$, $v_{z}$ are the Cartesian components of v in the external system. With this rotation, $b_{k}^{\prime }$ is renormalized to $b_{k\left(\mathrm{corr}\right)}^{\prime }$ so that $|b_{k\left(\mathrm{corr}\right)}^{\prime }|=1$. i.e.,

(6) $$b_{k\left(\mathrm{corr}\right)}^{\prime }=b_{k}^{\prime }/|b_{k}^{\prime }|≃[1-\frac{1}{2}\left(\right|b_{k}^{\prime }{|}^{2}-1)]b_{k}^{\prime }\;.$$

This is done to suppress roundoff errors characteristic of computer work (Note that $|b_{k}^{\prime }-b_{k\left(\mathrm{corr}\right)}^{\prime }|\ll 1$). If the bond vector $b_{k}$ does not undergo the rotation, on the other hand, we have $b_{k}^{\prime }=b_{k}$.

Then, the adoption of the next trial configuration $\left\{b_{N}^{\prime }\right\}$ is determined by the Metropolis method of importance sampling [26] on the basis of the total potential energies given by Equation (1) for $\left\{b_{N}^{\prime }\right\}$ and $\left\{b_{N}\right\}$. That is, $\left\{b_{N}^{\prime }\right\}$ is adopted as the next configuration with the (transition) probability $\tau (\left\{b_{N}^{\prime }\right\}|\left\{b_{N}\right\})$ defined as

(7) $$\tau \left(\left\{b_{N}^{\prime }\right\}|\left\{b_{N}\right\}\right)=\min\left(1,e^{-\beta \Delta U}\right)$$

with ΔU given by

(8) $$\Delta U=U\left(\left\{b_{N}^{\prime }\right\}\right)-U\left(\left\{b_{N}\right\}\right)=\frac{\alpha }{2}\left(\theta_{i}^{\prime \;2}+\theta_{j}^{\prime \;2}-\theta_{i}^{\;2}-\theta_{j}^{\;2}\right)\;$$

where $\theta_{i}^{\prime }$ (i=2, 3, ⋯, *N*) is the angle between $b_{i-1}^{\prime }$ and $b_{i}^{\prime }$, and $\theta_{1}^{\prime }$ the angle between $b_{N}^{\prime }$ and $b_{1}^{\prime }$. If $\left\{b_{N}^{\prime }\right\}$ is discarded, $\left\{b_{N}\right\}$ was again adopted as the next configuration. Through this MC algorithm, we sample one configuration at every $M_{\mathrm{nom}}$ (nominal) steps and $N_{s}$ configurations in total after an equilibration of $10^{4}\times M_{\mathrm{nom}}$ steps. $M_{\mathrm{nom}}$ is properly chosen to keep the mean number of (real) configurational changes at every $M_{\mathrm{nom}}$ (nominal) steps nearly equal to *N*. An ensemble of $N_{s}$ configurations so obtained is a mixture of configurations of all kinds of knots with the Boltzmann weight of *U*, which we call the mixed ensemble.

Following the procedure of Vologodskii et al. [27] and of ten Brinke and Hadziioannou [15] to distinguish the trefoil knot from the others by the use of the Alexander polynomial [23,28], we extract configurations of the trefoil knot (without distinguishing between the left- and right-handed knots) from the mixed ensemble and evaluate the ratio $f_{\mathrm{tref}.}$ of the number of the configurations of the trefoil knot to $N_{s}$. We note that the procedure on the basis of the Alexander polynomial cannot distinguish between the trefoil knot and, for the simplest example, the 8${}_{19}$ knot, as pointed out by ten Brinke and Hadziioannou [15]. However, effects of such complex knots may be regarded as negligibly small if any, as also pointed out by ten Brinke and Hadziioannou [15]. Further, we extract $N_{s}$ configurations of the trefoil knot from many mixed ensembles and construct a trefoil-knot ensemble.

Now, by the use of the trefoil-knot ensemble, the mean-square radius of gyration $R_{g}^{\;2}$ and scattering function P(k) as a function of the magnitude *k* of the scattering vector are evaluated. The quantity $R_{g}^{\;2}$ may be calculated from

(9) $$R_{g}^{\;2}=\langle \frac{1}{N}\sum_{i=1}^{N}{\left|S_{i}\right|}^{2}\rangle \;,$$

where 〈⋯〉 means the ensemble average and $S_{i}$ is the vector distance from the center of mass of the ring to the *i*th junction point, given by

(10) $$S_{i}=\sum_{j=1}^{i}b_{j}-\frac{1}{N}\sum_{j=1}^{N}\sum_{k=1}^{j}b_{k}$$

with $S_{0}=S_{N}$. Assuming that the KP ring has *N* identical isotropic point scatterers at each junction, P(k) may be calculated from

(11) $$P\left(k\right)=N^{-1}+2N^{-2}\sum_{i=1}^{N-1}\sum_{j=i+1}^{N}\langle \frac{\sin(kr_{ij})}{kr_{ij}}\rangle \;,$$

where $r_{ij}=\left|S_{j}-S_{i}\right|$ is the distance between the *i*th and *j*th junction points.

In practice, MC simulations have been carried out for the discrete KP rings of βα=0 (freely jointed), 0.3, 1, 3, and 10, with various values of *N*: N=10, 20, 50, 100, 200, 500, and 1000 for βα=0; N=10, 20, 50, 100, and 200 for βα=0.3 and 1; N=20, 50, 100, and 200 for βα=3; and N=50, 100, and 200 for βα=10. The values of $\lambda^{-1}/b$ for each βα are calculated from Equation (3) with Equation (2) as follows: $\lambda^{-1}/b=1$, 1.408, 2.575, 6.421, and 20.36 for βα=0, 0.3, 1, 3, and 10, respectively. Five independent trefoil-knot ensembles are constructed for each βα and *N* with $N_{s}=10^{5}$, except for the case of βα and N=1000. For that case, $N_{s}$ is set equal to $10^{4}$. All numerical work has been done by the use of a personal computer with an Intel Core i7-3770 CPU. A source program coded in C has been compiled by the GNU C compiler version 4.8.5 with real variables of double precision. For a generation of pseudorandom numbers, the subroutine package MT19937 supplied by Matsumoto and Nishimura [29] has been used instead of the subroutine RAND included in the standard C library.

## 3. Results and Discussion

### 3.1. Fraction of the Trefoil Knot

The ratio $f_{\mathrm{tref}.}$ of the number of configurations of the trefoil knot in a given mixed ensemble to the total number $N_{s}$ of configurations in the ensemble is evaluated. The values of $f_{\mathrm{tref}.}$ and its statistical error are given in the second column of Table 1 as the mean and standard deviation, respectively, of five independent MC results for given values of βα and *N*.

**Figure 1:**
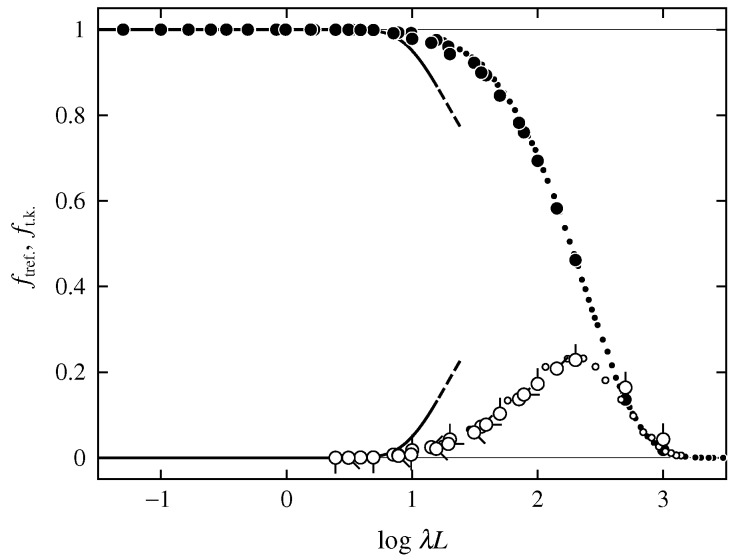
Plots of $f_{\mathrm{tref}.}$ and $f_{t.k.}$ against logλL. The large open circles represent the Monte Carlo (MC) values of $f_{\mathrm{tref}.}$ for the discrete Kratky–Porod (KP) ring with βα=0 (pip up); 0.3 (pip right-up); 1 (pip right); 3 (pip right-down); and 10 (pip down). The small open circles represent the MC values of $f_{\mathrm{tref}.}$ for the Gaussian ring obtained by Tsurusaki and Deguchi [16]. The large and small closed circles represent the MC values of $f_{t.k.}$ for the discrete KP ring obtained in the previous study [7] and those for the freely jointed ring obtained by Moore et al. [20], respectively. The lower and upper curves represent the theoretical values for $f_{\mathrm{tref}.}$ and $f_{t.k.}$, respectively, calculated on the assumption that the mixed ensemble includes only the most-probable configurations of the trefoil and trivial knots (see text).

Figure 1 shows plots of $f_{\mathrm{tref}.}$ against the logarithm of the reduced contour length λL, defined as the total contour length L=Nb measured in units of $\lambda^{-1}$. The large open circles represent the MC values of the discrete KP ring for βα=0 (pip up), 0.3 (pip right-up), 1 (pip right), 3 (pip right-down), and 10 (pip down). The MC values of the Gaussian ring obtained by Tsurusaki and Deguchi [16] are also plotted (with the number of bonds of the Gaussian ring converted properly to λL) by small open circles. We note that Tsurusaki and Deguchi adopted the procedure for extracting configurations of the trefoil knot proposed by themselves [16,17] using not only the Alexander polynomial, but also the Vassiliev invariants [30] of degree 2 and 3. Although the values of the discrete KP ring are slightly larger than those for the Gaussian ring for λL≳200 due to the difference in the model, the data points of $f_{\mathrm{tref}.}$ for the discrete KP rings with various values of βα (or $\lambda^{-1}$), along with those for the Gaussian ring, may be regarded as forming a single composite curve. This indicates that $f_{\mathrm{tref}.}$ is a function only of λL. With increasing λL, $f_{\mathrm{tref}.}$ first increases from zero in the range of λL≳10, and then decreases to zero after passing through a maximum at λL≃200.

For comparison, the ratio $f_{t.k.}$ of the number of configurations of the trivial knot in a given mixed ensemble to *N* are also plotted against logλL in Figure 1. The large and small closed circles represent the MC values of the discrete KP ring (with various values of βα) reproduced from Figure 2 of Ref. [7] and those for the freely jointed ring obtained by Moore et al. [20], respectively. The ratio $f_{t.k.}$ is also a function only of λL is almost equal to unity for λL≲10, and decreases monotonically to zero with increasing λL for λL≳10, as pointed out in the previous study [7]. The important point is that with increasing λL, both the increase of $f_{\mathrm{tref}.}$ from zero and decrease of $f_{t.k.}$ from unity begin at λL≃10.

For small λL, ring polymers become stiff and short and then configurations of complex knots may be rarely (or never) realized. A given mixed ensemble for λL≲10 may therefore be regarded asymptotically as including only the most-probable configurations of the trivial and trefoil knots. In such a situation, there may hold the relation,

(12) $$f_{t.k.}+f_{\mathrm{tref}.}=1$$

The most-probable configuration of the KP ring of the trivial knot with reduced contour length λL (and without chain sickness) is, of course, the circle of radius λL/2π, and its configurational energy $U_{0,t.k.}$ may be given by $U_{0,t.k.}=\pi^{2}/\beta \lambda L$ [32]. Additionally, the most-probable configuration of the KP ring of the trefoil knot with reduced contour length λL is the double circle of radius λL/4π (two circles of radius of λL/4π completely overlapping each other), and then its configurational energy $U_{0,\mathrm{tref}.}$ may be given by $U_{0,\mathrm{tref}.}=4\pi^{2}/\beta \lambda L$. In the above-mentioned situation, $f_{\mathrm{tref}.}$ may be expressed by the use of $U_{0,t.k.}$ and $U_{0,\mathrm{tref}.}$ as follows:

(13) $$f_{\mathrm{tref}.}=\frac{e^{-\beta U_{0,\mathrm{tref}.}}}{e^{-\beta U_{0,t.k.}}+e^{-\beta U_{0,\mathrm{tref}.}}}=\frac{e^{-4\pi^{2}/\lambda L}}{e^{-\pi^{2}/\lambda L}+e^{-4\pi^{2}/\lambda L}}\;$$

In Figure 1, the theoretical values of $f_{\mathrm{tref}.}$ calculated from Equation (13), and those of $f_{\mathrm{tref}.}$ calculated from Equation (12) with Equation (13) are plotted by the lower and upper curves, respectively. It is seen that the theoretical results may qualitatively explain both the increase of $f_{\mathrm{tref}.}$ and the decrease of $f_{t.k.}$ in the range of λL≲10.

### 3.2. Mean-Square Radius of Gyration

The mean-square radius of gyration $R_{g}^{\;2}$ is calculated from Equation (9) with Equation (10). The values of $R_{g}^{\;2}/Nb^{2}$ and its statistical error are given in the third column of Table 1 as the mean and standard deviation, respectively, of five independent MC results for given values of βα and *N*.

**Figure 2:**
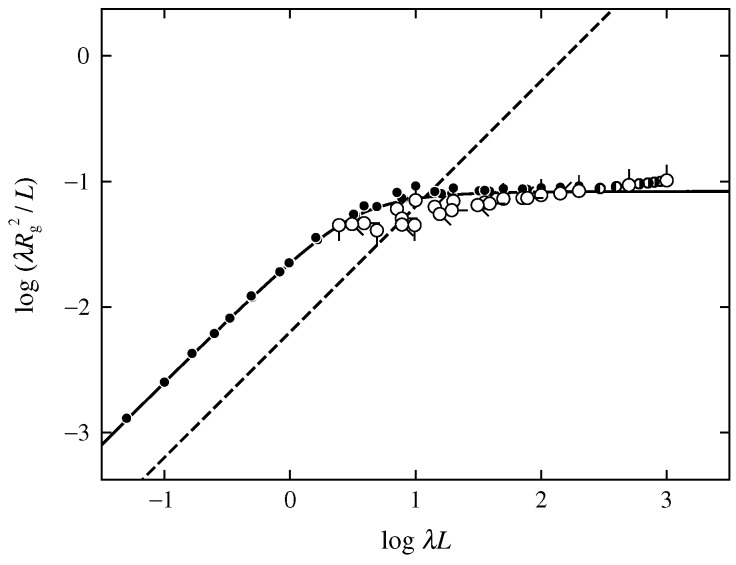
Double-logarithmic plots of $\lambda R_{g}^{\;2}/L$ against λL. The open circles represent the MC values for the discrete KP ring of the trefoil knot, various directions of the pip having the same meaning as those in Figure 1. The half-filled circles represent the MC values for the freely jointed ring obtained by Dobay et al. [19] (left-half filled) and by Moore et al. [20] (right-half filled). The dashed straight line represents the theoretical values of the double circle. The closed circles represent the MC values for the discrete KP ring (with various values of βα) obtained in the previous study [7], and the solid curve represents the KP theoretical values (without considering the effects of the topological constraints) [5,11,31].

Figure 2 shows double-logarithmic plots of $\lambda R_{g}^{\;2}/L$ against λL. The open circles represent the MC values for the discrete KP ring of the trefoil knot, various directions of pips having the same meaning as those in Figure 1. The half-filled circles represent the MC values for the freely jointed rings of the trefoil knot obtained by Dobay et al. [19] (left-half filled) and by Moore et al. [20] (right-half filled). We note that for the extraction of the configurations of the trefoil knot, Moore et al. adopted the above-mentioned procedure by Deguchi and Tsurusaki [16,17], and Dobay et al. did the procedure on the basis of the HOMFLY polynomials [23]. The dashed straight line represents the theoretical values of the double circle (most-probable configuration of the KP ring of the trefoil knot in the limit of λL→0), calculated from $R_{g}^{\;2}=L^{2}/16\pi$. For comparison, the MC data for the discrete KP ring of the trivial knot are also plotted, reproduced from Figure 3 of Ref. [7] (represented by the closed circles). Additionally, the theoretical values for the (continuous) KP ring without the topological constraints (phantom KP ring), corresponding to the values for the mixed ensemble, calculated from [5,11,31]

(14) $$\begin{array}{ccc} \frac{\lambda R_{g}^{\;2}}{L} & = & \frac{\lambda L}{4\pi^{2}}{[1-0.1140\lambda L-0.0055258\left(\lambda L\right)}^{2}\\ & & \;+0.0022471{\left(\lambda L\right)}^{3}-0.00013155{\left(\lambda L\right)}^{4}]\;\mathrm{for}\lambda L\leq 6\\ & = & \frac{1}{12}\left\{1-\frac{7}{6\lambda L}-0.025\exp\left[-0.01{\left(\lambda L\right)}^{2}\right]\right\}\;\mathrm{for}\lambda L>6\; \end{array}$$

is represented by the solid curve. We note that the MC values for the mixed ensemble were obtained in the previous study [7], which agree almost completely with the theoretical values calculated from Equation (14).

The data points for the discrete KP ring of the trefoil knot in the range of N≥100 for each βα, along with those for the Gaussian ring of the trefoil knot, seem to form a single composite curve, although the data points for the discrete KP ring in the range of N≤50 are scattered because of chain discreteness. This indicates that $\lambda R_{g}^{\;2}/L$ for the KP ring of the trefoil knot is also a function only of λL, as in the case of the KP ring of the trivial knot [7]. The single composite curve seems to increase along the dashed straight line and then deviate downward from the line for λL≳10 with increasing λL. Furthermore, it is seen that the single composite curve for the trefoil knot increases monotonically with increasing λL for λL≳10 and crosses over the KP theory curve (solid curve) at λL≃200, while the data points for the trivial knot only deviate upward from the KP theory curve with increasing λL for λL≳10 without crossing over the KP theory curve.

**Figure 3:**
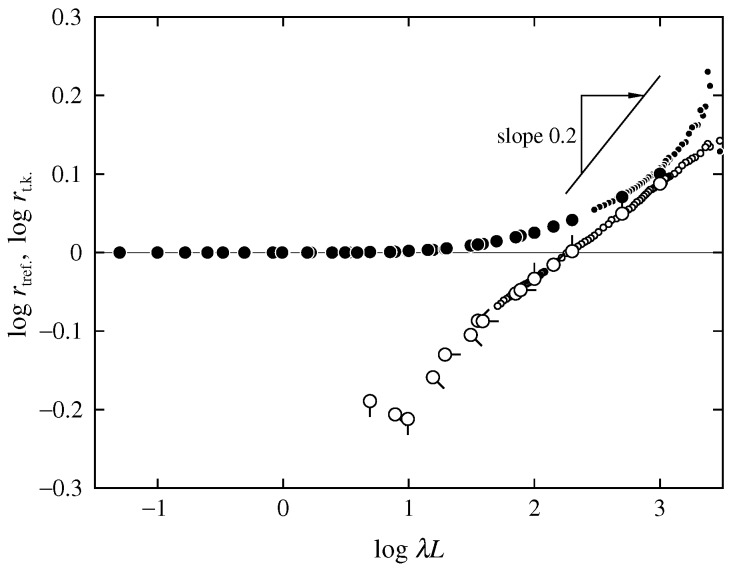
Double-logarithmic plots of $r_{\mathrm{tref}.}$ and $r_{t.k.}$ against λL. The large open circles represent the MC values of $r_{\mathrm{tref}.}$ for the discrete KP ring, various directions of the pip having the same meaning as those in Figure 1. The small open circles represent the MC values of $r_{\mathrm{tref}.}$ for the freely jointed ring obtained by Moore et al. [20]. The large and small closed circles represent the MC data of $r_{t.k.}$ for the discrete KP ring obtained in the previous study [7] and those for the freely jointed ring obtained by Moore et al. [20], respectively.

Such a situation may be realized more clearly from a comparison between the behavior of the ratios $r_{\mathrm{tref}.}$ and $r_{t.k.}$ of $R_{g}^{\;2}$ for the trefoil and trivial knots, respectively, to $R_{g}^{\;2}$ without the topological constraints. Figure 3 shows double-logarithmic plots of $r_{\mathrm{tref}.}$ and $r_{t.k.}$ against λL. The large open circles represent the MC values of $r_{\mathrm{tref}.}$ for the discrete KP ring with N≥100 for each βα, calculated from the MC values of $R_{g}^{\;2}/Nb^{2}$ for the trefoil knot given in Table 1, and those for the phantom KP ring given in Table 2 of Ref. [7], various pip directions having the same meaning as in Figure 1. We note that the data for N≤50 are omitted because of the effects of chain discreteness mentioned above. The small open circles represent the MC values of $r_{\mathrm{tref}.}$ for the freely jointed ring obtained by Moore et al. [20]. The large and small closed circles represent the MC data of $r_{t.k.}$ for the discrete KP ring reproduced from Figure 4 of Ref. [7] and those for the freely jointed ring obtained by Moore et al. [20], respectively.

The asymptotic value of $r_{\mathrm{tref}.}$ in the limit of λL→0 is 1/4 (calculated from the relations $R_{g}^{\;2}=L^{2}/16\pi$ for the trefoil knot and $R_{g}^{\;2}=L^{2}/4\pi$ for the trivial knot in this limit). The ratio $r_{\mathrm{tref}.}$ may be considered to increase monotonically from the asymptotic value 1/4 and become larger than unity with increasing λL, while $r_{t.k.}$ increases monotonically from unity with increasing λL. In the limit of λL→∞, the $R_{g}^{\;2}$ of both the trefoil and trivial knot may be considered to be proportional to ${\left(\lambda L\right)}^{1.2}$ [18]. Considering the fact that $R_{g}^{\;2}$ without the topological constraints is proportional to λL in this limit [1,33,34], there hold relations $r_{\mathrm{tref}.}\propto {\left(\lambda L\right)}^{0.2}$ and $r_{t.k.}\propto {\left(\lambda L\right)}^{0.2}$ in the same limit. Unfortunately, however, the present data as well as the data by Moore et al.—both up to $O[{\left(\lambda L\right)}^{3}]$—seem to be far from the limit of λL→∞, and thus the validity of the predicted asymptotic behavior cannot be confirmed.

### 3.3. Scattering Function

Finally, we give the results for the scattering function P(k) as a function of the magnitude *k* of the scattering vector. The function P(k) is calculated from Equation (11) for the discrete KP ring, with n=200 for each βα, the corresponding values of λL being 200, 142.0, 77.67, 31.15, and 9.823 for βα=0, 0.3, 1, 3, and 10, respectively.

**Figure 4:**
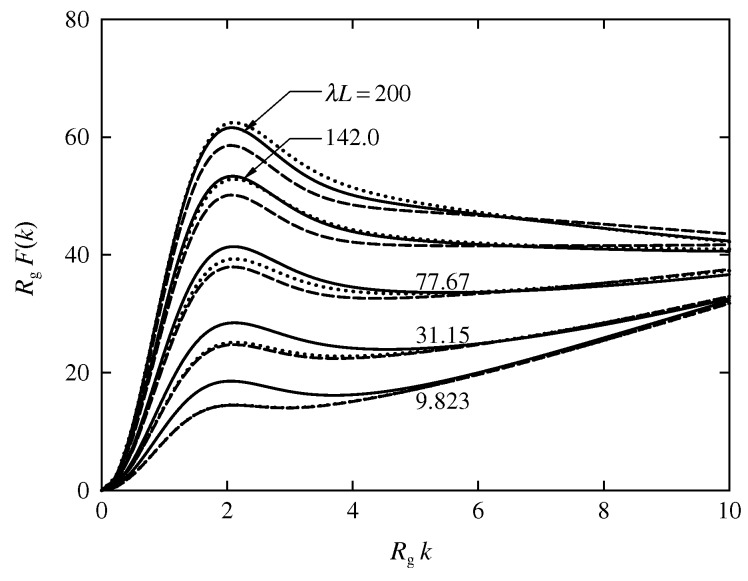
Plots of $R_{g}F\left(k\right)$ against $R_{g}k$ for the discrete KP rings of the trefoil knot (solid); of the trivial knot (dashed) [8]; and without the topological constraints (dotted) [8], with the indicated values of λL.

Figure 4 shows plots of $R_{g}F\left(k\right)$ against $R_{g}k$ (the reduced Kratky plot) for the discrete KP rings, where F(k) is the Kratky function defined by $F\left(k\right)=Lk^{2}P\left(k\right)$ [5]. The solid, dashed, and dotted curves represent the MC values for the trefoil knot, for the trivial knot, and without the topological constraints, respectively, with the indicated values of λL, the latter two kinds of curves being reproduced from Figure 1 of Ref. [8]. The plots for the trefoil knot have a peak in the range of $R_{g}k≲3$, as in the cases of the discrete KP ring of the trivial knot and that without the topological constraints. The values of $R_{g}F\left(k\right)$ for the trefoil knot are always larger than those for the trivial knot in the range of $R_{g}k≲6$, irrespective of λL. On the other hand, in the same range of $R_{g}k$, $R_{g}F\left(k\right)$ for the trefoil knot is larger than $R_{g}F\left(k\right)$ without the topological constraints for λL≤77.67, while it is nearly equal to or smaller than $R_{g}F\left(k\right)$ without the topological constraints for λL≥142.0. Such behavior of $R_{g}F\left(k\right)$ for the trefoil knot with λL=200 is consistent with the behavior of the Gaussian ring [21].

## 4. Concluding Remarks

The mean-square radius of gyration $R_{g}^{\;2}$ and scattering function P(k) with *k* being the magnitude of the scattering vector have been evaluated for the KP ring of the trefoil knot by MC simulations. The behavior of $R_{g}^{\;2}$ and P(k) as functions of the reduced contour length λL—defined as the total contour length *L* measured in units of the stiffness parameter $\lambda^{-1}$—has been clarified herein. A comparison has been made of the present results with the previous results for the KP ring of the trivial knot and for the phantom KP ring without topological constraints.

The double-logarithmic plots of $\lambda R_{g}^{\;2}/L$ against λL have been shown to increase along the straight line of slope unity, representing the values of the double circle (most-probable configuration of the KP ring of the trefoil knot in the limit of λL→0) and then to deviate downward from the line, crossing over the theoretical values of the phantom KP ring. The reduced Kratky plots for the KP ring of the trefoil knot have been shown to have a characteristic peak, as in the cases of the KP ring of the trivial knot and the phantom KP ring. It has also been found that the height of the peak for the trefoil knot is larger than that for the trivial knot, irrespective of λL and for the phantom KP ring with small λL, while it is smaller than the height of the peak for the phantom KP ring with large λL.

Finally, we make brief comments on the following two points related to the present work. The first concerns (intramolecular) excluded-volume effects on semiflexible ring polymers. Even for semiflexible polymers, the excluded volume effects become remarkable if *M* (or λL) is very large. The effects must be treated in the quasi-two-parameter (or Yamakawa–Shimada–Stockmayer) scheme [5]. In this scheme, we should first clarify the behavior of the unperturbed chain dimension ($R_{g}^{\;2}$ at Θ) as a function of *M* (or λL), and then examine the behavior of the perturbed chain dimension ($R_{g}^{\;2}$ in good solvents) or the corresponding expansion factor (gyration-radius expansion factor) as a function of *M* (or λL) and the excluded-volume strength (or the binary cluster integral between segments). This is also the case with semiflexible ring polymers. The present and previous [7,8,9] studies may be regarded as preliminary. In future work, we hope to examine the excluded volume effects on ring polymers on the basis of the present results. Next, we will discuss the some applications that may arise from the present work. Recently, the manipulation of a single biological polymer by an external field seems to have become important in the field of biology. It has been shown that the stretching behavior of semiflexible (linear) polymers by an external field is largely affected by the chain stiffness [35]. The stretching behavior of semiflexible rings (e.g., circular DNA) may also be considered to be affected by their knot types and chain stiffness, due to the difference in the repeat-unit distribution around the center of mass (or in $R_{g}^{\;2}$ and P(k), as shown in Figure 2 and Figure 4, respectively) between the rings of different knot types. Future study investigating the effects of intramolecular topological constraints on the stretching behavior of semiflexible rings is also of interest.

## Figures and Tables

**Table 1:** Values of $f_{\mathrm{tref}.}$ and $R_{g}^{\;2}/Nb^{2}$.

*N*	$10^{2}f_{\mathrm{tref}.}$ (Error %)	$R_{g}^{\;2}/{Nb}^{2}$ (Error %)
βα=0 ($\lambda^{-1}/b=1$)		
10	1.7${}_{6}$ (0.3)	0.0708${}_{8}$ (0.1)
20	4.2${}_{9}$ (0.4)	0.0698${}_{2}$ (0.1)
50	10.3${}_{1}$ (0.3)	0.0728${}_{0}$ (0.1)
100	17.2${}_{7}$ (0.3)	0.0778${}_{8}$ (0.1)
200	22.8${}_{5}$ (0.3)	0.0841${}_{1}$ (0.1)
500	16.4${}_{0}$ (0.1)	0.0936${}_{0}$ (0.1)
1000	4.3${}_{1}$ (0.1)	0.102${}_{0}$ (0.1)
βα=0.3 ($\lambda^{-1}/b=1.408$)		
10	0.7${}_{4}$ (0.3)	0.0852${}_{7}$ (0.1)
20	2.4${}_{8}$ (0.3)	0.0884${}_{5}$ (0.1)
50	7.2${}_{9}$ (0.2)	0.0957${}_{3}$ (0.2)
100	13.6${}_{8}$ (0.2)	0.104${}_{0}$ (0.1)
200	20.8${}_{4}$ (0.1)	0.113${}_{2}$ (0.1)
βα=1 ($\lambda^{-1}/b=2.575$)		
10	0.0${}_{7}$ (0.3)	0.119${}_{9}$ (0.1)
20	0.6${}_{2}$ (0.2)	0.130${}_{4}$ (0.2)
50	3.2${}_{4}$ (0.2)	0.152${}_{0}$ (0.1)
100	7.7${}_{5}$ (0.2)	0.171${}_{6}$ (0.1)
200	14.7${}_{7}$ (0.2)	0.190${}_{0}$ (0.0)
βα=3 ($\lambda^{-1}/b=6.421$)		
20	0.0${}_{2}$ (0.2)	0.285${}_{8}$ (0.0)
50	0.4${}_{5}$ (0.2)	0.283${}_{1}$ (0.1)
100	2.1${}_{0}$ (0.2)	0.343${}_{7}$ (0.1)
200	5.9${}_{3}$ (0.2)	0.404${}_{9}$ (0.1)
βα=10 ($\lambda^{-1}/b=20.36$)		
50	0.0${}_{2}$ (0.3)	0.914${}_{2}$ (0.0)
100	0.0${}_{9}$ (0.4)	0.830${}_{2}$ (0.2)
200	0.7${}_{6}$ (0.3)	0.914${}_{7}$ (0.1)
